# Glial Plasticity

**DOI:** 10.1155/2015/723891

**Published:** 2015-08-05

**Authors:** Tomas C. Bellamy, Anna Dunaevsky, H. Rheinallt Parri

**Affiliations:** ^1^School of Life Sciences, University of Nottingham Medical School, Nottingham NG7 2UH, UK; ^2^Developmental Neuroscience, Munroe-Meyer Institute, University of Nebraska Medical Center, 985960 Nebraska Medical Center, Omaha, NE 68198-5960, USA; ^3^School of Life and Health Sciences, Aston University, Birmingham B4 7ET, UK

Over the last few decades, our understanding of the roles of glial cells in the central nervous system has been transformed. There is now a clear consensus that all classes of glia (astrocytes, oligodendrocytes, microglia, and various other progenitors and specialized cells) can detect and respond to a wide range of neurotransmitters, hormones, cytokines, and trophic factors and thereby play an active, signaling role in neurophysiology. To date, much of the focus of glial signaling has been on how glia can influence the function of the neuronal network with the associated impact on information processing and, ultimately, behavior. In particular, the contribution of bidirectional communication between neurons and glia to the regulation of synaptic plasticity has been extensively studied.

The papers collected in this special issue focus on a related, but distinct, question: can glia themselves exhibit activity-dependent plasticity? The reviews and experimental papers present the evidence that glia do indeed have the capacity to respond dynamically to a wide range of stimuli with persistent changes in signal transduction, morphology, and homeostasis.

In “Plasticity of Neuron-Glial Transmission: Equipping Glia for Long-Term Integration of Network Activity,” W. Croft et al. review the current evidence for plasticity in neuron-glial communication and speculate on the implication of differences in induction paradigms from synaptic plasticity for the computational properties of glial signaling.

In “Glutamatergic Transmission: A Matter of Three,” Z. Martínez-Lozada and A. Ortega focus on the consequences of glutamate receptor activation for astroglial physiology. By identifying the downstream targets engaged by glutamatergic signalling, the authors show how neurons can shape transcriptional and translational regulation in glia to tailor transmitter clearance and recycling to meet synaptic demands.

Remaining with regulation of intracellular signaling in astrocytes, N. Komin et al. present an analysis of the impact of variation in uptake of calcium into endoplasmic reticulum stores on cytoplasmic calcium oscillations in “Multiscale Modeling Indicates That Temperature Dependent [Ca^2+^]_*i*_ Spiking in Astrocytes Is Quantitatively Consistent with Modulated SERCA Activity.” The results of the modelling study illustrate the striking sensitivity of intracellular calcium dynamics to changes in SERCA activity with implications both for interpretation of experimental results at nonphysiological temperatures and for prediction of calcium signal kinetics in vivo.

In “Fractalkine Signaling and Microglia Functions in the Developing Brain,” I. Arnoux and E. Audinat review the effects of fractalkine receptor activation on microglial function. The review discusses the control of microglial recruitment to sites of developing synapses, where the glia contribute to synapse elimination but also support neuronal survival, axon outgrowth, and laminar positioning. Breakdown of this mechanism for bidirectional communication is also shown to be implicated in neurodevelopmental disorders.

A focus on the plasticity of well-known “housekeeping” roles of astrocyte potassium and glutamate uptake is the subject of “Activity-Dependent Plasticity of Astroglial Potassium and Glutamate Clearance.” Here, G. Cheung et al. review the evidence for and the characteristics of short and long term changes in uptake and clearance and their relationship with neuronal synaptic plasticity.

Although there is ample evidence for the role of astrocytes in regulating synaptic function, studies of the role of astrocytes in behavior and learning in particular are scant. In the manuscript “Motor-Skill Learning Is Dependent on Astrocytic Activity,” R. Padmashri et al. use genetic (IP_3_R2 mutant mouse) and pharmacological approaches to demonstrate that motor-skill learning is impaired in mice with attenuated astrocytic activity. Moreover, they show that astrocytic activity is necessary for normal LTP in the primary motor cortex and that administration of the gliotransmitter D-serine partially rescues LTP in slices with reduced Ca^2+^ signaling and reverses the learning impairment in the motor-skill learning task. These results provide evidence that normal astrocytic Ca^2+^ signaling during a reaching task is necessary for motor-skill learning.

Prenatal exposure of the developing brain to various types of environmental stress increases susceptibility to neuropsychiatric disorders. While effects of antenatal exposure to corticosteroids on neuronal development have been previously described, the impact on astrocytes has not been studied extensively. In the manuscript “Astroglial Plasticity Is Implicated in Hippocampal Remodelling in Adult Rats Exposed to Antenatal Dexamethasone,” V. H. Shende et al. demonstrate long lasting effects of dexamethasone on hippocampal astrocytes in offspring whose mothers were exposed during pregnancy. Interestingly, the deficits were in astrocytic branch development rather than number of cells indicating a role in astrocytic morphological plasticity.

H. R. Parri et al. review the roles of astrocytes in the barrel cortex in “Astrocyte and Neuronal Plasticity in the Somatosensory System.” Here the physiological roles of two major forms of neuronal plasticity (termed homeostatic and coding plasticity) are well understood, but the recent discovery of several forms of astroglial plasticity suggests that these cells also have the capacity to play a computational role in experience-dependent plasticity.

Collectively, these studies demonstrate that glial cells express a wide range of plasticity, which could impact on many aspects of neurophysiology. Clearly, the computational potential of these cells deserves further scrutiny.

*Tomas C. Bellamy* *Tomas C. Bellamy* *Anna Dunaevsky* *Anna Dunaevsky* *H. Rheinallt Parri* *H. Rheinallt Parri*

